# Targeting TLR2 to Overcome Autophagy‐Dependent Chemoresistance in Gastric Cancer: Integrating Molecular Docking, Mechanistic Validation, and Therapeutic Evaluation

**DOI:** 10.1002/jbt.71137

**Published:** 2026-11-11

**Authors:** Kuanheng Zheng, Xinjian Lei, Bin Lin, Qianhong Yang, Cong He, Jin Gao, Feng Li

**Affiliations:** ^1^ Department of Gastrointestinal Surgery Zone 2 Ningde Clinical Medical College of Fujian Medical University Ningde City Fujian China; ^2^ Department of Gastrointestinal Surgery Ningde Municipal Hospital Ningde City Fujian China

**Keywords:** autophagy, chemoresistance, gastric cancer, molecular docking, TLR2

## Abstract

Chemoresistance remains a major barrier to effective treatment of gastric cancer (GC), particularly in advanced disease, where cytotoxic chemotherapy often yields limited and transient responses. The present study provides integrated computational and experimental evidence that targeting TLR2 using repurposed small‐molecule inhibitors can overcome autophagy‐dependent chemoresistance in gastric adenocarcinoma cells. A library comprising 1555 FDA‐approved drugs was sourced from the DrugBank database, virtual screening was executed, and two compounds, Nilotinib (DB04868) and Lomitapide (DB08827), achieved affinities ≤ −10 kcal/mol. Chemoresistant GC cells were developed, and experimental assessment was performed using cell proliferation assay, apoptosis assay, real‐time PCR, and western blot analysis. Nilotinib and Lomitapide significantly reduced the proliferation but increased the apoptosis of chemoresistant GC cells. Additionally, Nilotinib and lomitapide reduced the mRNA and protein levels of autophagy‐related factors, *LC3B*, *Beclin‐1*, and *p62/SQSTM1*, and activated transcription factors in chemoresistant GC cells. In conclusion, our results suggest that combining TLR2‐targeted agents with standard chemotherapy could be a promising strategy to overcome refractory disease in GC.

## Graphical abstract

Overcoming chemoresistance in gastric cancer by targeting TLR2.

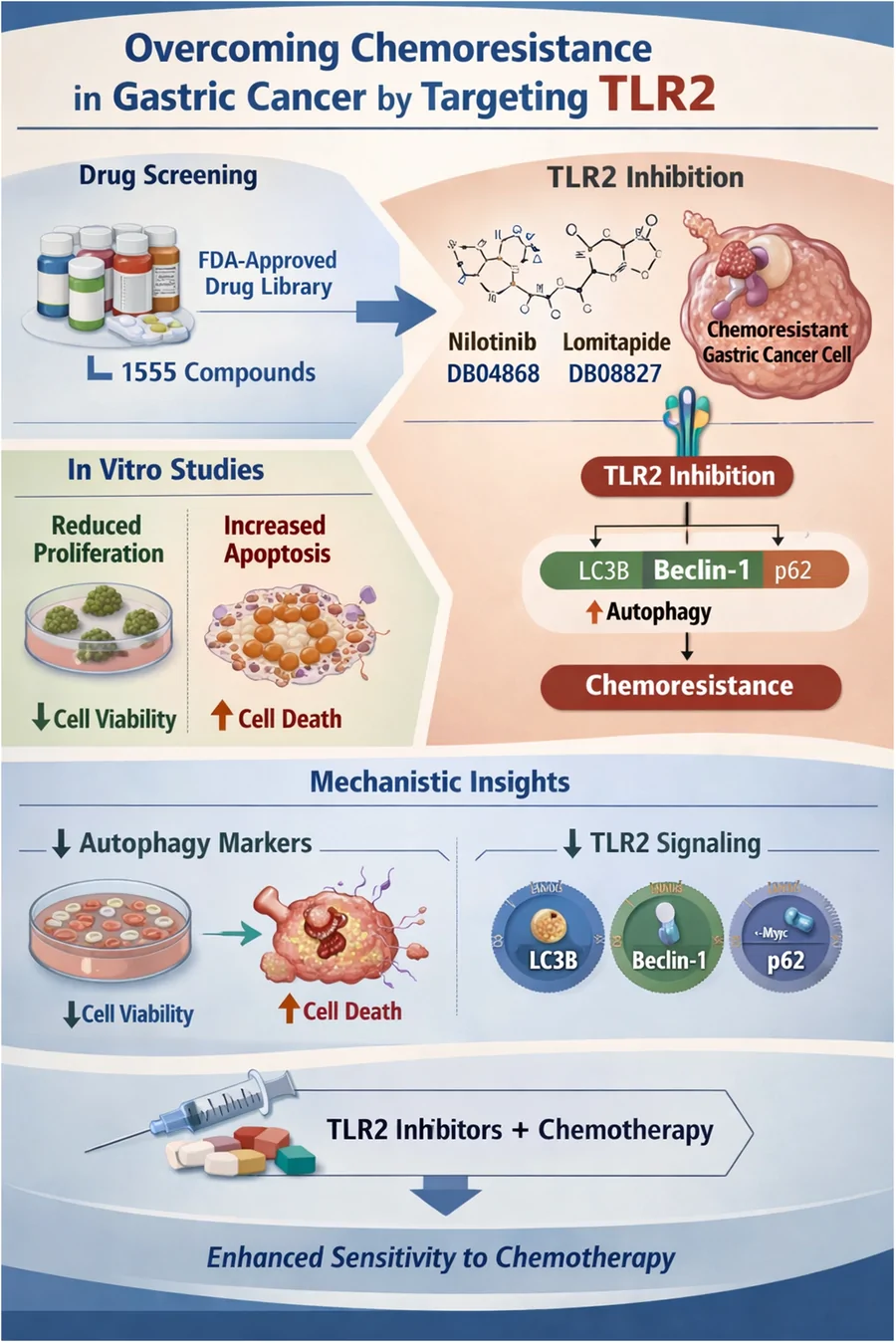

## 1 Introduction

Gastric cancer (GC) remains one of the leading causes of cancer‐related mortality worldwide due to late diagnosis, high rates of recurrence, and especially the frequent resistance to systemic chemotherapy [1]. Although platinum‐ and fluoropyrimidine‐based regimens constitute the main treatment for advanced GC, the efficacy treatments is often limited by chemoresistance. Chemoresistance significantly reduces therapeutic efficacy by enabling tumor cells to evade cytotoxic effects and promote survival pathways, highlighting the urgent need for novel molecular targets and therapeutic strategies in GC management [2]. Therefore, elucidating the molecular mechanisms underlying chemoresistance and identifying targets to restore drug sensitivity are critical priorities for improving clinical outcomes in GC.

Autophagy is an evolutionarily conserved lysosomal degradation process that plays a dual role in cancer development and therapy. It acts either as a pro‐survival mechanism that facilitates tumor cell adaptation under stress or as a programmed cell death pathway depending on cellular context and signaling cues [3, 4]. Basal autophagy usually contributes to cellular homeostasis and tumor suppression in early carcinogenesis stages, while established tumors can employ autophagy as a cytoprotective mechanism to survive metabolic stress and chemotherapy‐induced damage [5]. Increasing evidence supports the role of autophagy in chemoresistance in GC by facilitating cellular adaptation to cytotoxic stress, inhibiting apoptosis, and sustaining tumor cell viability making it a critical target for overcoming chemoresistance in GC [6, 7]. Consequently, targeting autophagy‐associated signaling pathways has emerged as a promising strategy to enhance chemosensitivity and overcome treatment resistance.

Toll‐like receptor 2 (TLR2) is a key pattern recognition receptor of the innate immune system that has recently gained attention for its non‐canonical roles in tumor biology [8, 9, 10]. It is expressed on both immune and tumor cells and signals through MyD88‐dependent signaling and NF‐κB activation [10, 11]. Contrary to its pro‐inflammatory functions, accumulating evidence shows that aberrant TLR2 signaling has been implicated in cancer cell proliferation, metabolic reprogramming, survival, inflammation‐driven tumor progression, and therapy resistance across several malignancies. For example, elevated TLR2 expression in GC has been reported to correlate with advanced disease features and enhanced metabolic shifts through the TLR2‐SOD2 axis, resulting in tumor progression and poor prognosis [12]. Moreover, studies in other cancer types have demonstrated that TLR2 activation can promote survival signaling and chemotherapy resistance, underscoring the TLR2 signaling as a potential target to sensitize tumors to cytotoxic agents [11, 13]. Notably, TLR2 activation has been shown to regulate autophagy through downstream pathways involving NF‐κB, MAPK, and PI3K/AKT/mTOR signaling [14, 15], suggesting a potential link between TLR2 signaling and autophagy‐dependent chemoresistance. However, the functional relevance of TLR2‐mediated autophagy in GC and its therapeutic target potential remains to be understood.

In this regard, integrating computational and experimental approaches can provide a powerful framework for therapeutic target discovery. Furthermore, preclinical therapeutic evaluation enables assessment of synergistic effects between TLR2 targeting and conventional chemotherapeutic agents. Molecular docking and in silico screening can identify candidate TLR2 inhibitors with favorable binding characteristics. Targeted modulation of TLR2, particularly through rational design of small‐molecule antagonists, could represent a promising strategy to reverse autophagy‐dependent resistance mechanisms. Previous works using structure‐based virtual screening have identified small molecules capable of binding the TLR2 and attenuating its downstream signaling, have demonstrated the feasibility of such approaches [16, 17, 18]. In this study, we investigated the role of TLR2 in autophagy‐dependent chemoresistance in GC by combining molecular docking analyses and mechanistic validation, with the aim of establishing TLR2 as a novel target for overcoming autophagy‐mediated chemoresistance in GC.

## 2 Materials and Methods

### 2.1 Retrieval and Preparation of TLR2‐TLR1 Structure and Binding Pocket Definition

The crystal structure of the TLR2‐TLR1 heterodimer bound to the lipopeptide agonist, PAM (Pam3CSK4), was obtained from the Protein Data Bank (PDB ID: 2Z7X) [19]. This structure was selected for its high resolution (2.1 Å) and clear depiction of the functionally relevant dimer interface essential for TLR2 activation [19]. The protein was prepared for docking using AutoDockTools (version 1.5.7), which involved removal of non‐essential water molecules and ligands, addition of polar hydrogens, and assignment of Gasteiger charges [20]. The binding pocket was defined at the TLR2‐TLR1 dimerization interface, targeting residues critical for ligand recognition and signaling (e.g., Phe325, Tyr326, Leu328 in TLR2, and analogous sites in TLR1) [21]. Grid parameters were configured as follows: center coordinates (*x* = −29.603, *y* = −3.535, *z* = 25.483), grid size (*x* = 46.296 Å, *y* = 35.830 Å, *z* = 48.396 Å), and a default grid spacing of 0.375 Å to encompass the interface and potential inhibitory binding sites.

### 2.2 Virtual Screening of FDA‐Approved Drug Library

A library comprising 1555 FDA‐approved drugs was sourced from the DrugBank database in SDF format [22] and converted to PDBQT using Open Babel (version 3.1.1) [23]. Virtual screening was executed with AutoDock Vina (version 1.1.2) [24] on a computational cluster, employing an exhaustiveness parameter of 8 and the default Vina scoring function for binding affinity estimation. Each ligand was docked into the defined pocket, and poses were ranked by predicted affinity (kcal/mol), with more negative values indicating stronger binding. Post‐docking analysis included visual inspection of top poses using Discovery Studio Visualizer (version 21.1.0) [25] and ChimeraX (version 1.7) [26] to evaluate interactions such as hydrogen bonds and hydrophobic contacts with key residues. Compounds with docking affinities ≤ −10 kcal/mol were prioritized as potential TLR2 antagonists, based on their superior binding scores and poses disrupting the dimer interface. The top‐ranking candidates fulfilling these criteria were selected for further evaluation using molecular dynamics simulations.

### 2.3 Molecular Dynamics Simulations

MD simulations were performed on the selected ligand‐TLR2 complexes using GROMACS (version 2023.3) [27] with the CHARMM36 force field [28]. Ligand parameters and topologies were generated using the SwissParam web server [29]. Each complex was solvated in a dodecahedral box with TIP3P water [30], neutralized with Na^+^/Cl^−^ ions to 0.15 M concentration [31], and energy‐minimized using the steepest descent algorithm (5000 steps, tolerance: 1000 kJ/mol/nm). Equilibration involved NVT (100 ps, 300 K, V‐rescale thermostat) followed by NPT (100 ps, 1 bar, Parrinello−Rahman barostat). Production runs lasted 100 ns each with a 2 fs timestep, LINCS constraints for bonds, and PME for long‐range electrostatics [32]. Trajectories were analyzed for stability using root‐mean‐square deviation (RMSD), root‐mean‐square fluctuation (RMSF), and hydrogen bond occupancy in GROMACS tools.

### 2.4 Binding Free Energy and Per‐Residue Energy Decomposition

Binding free energies were estimated with the molecular mechanics/generalized born surface area (MM‐GBSA) end‐point method implemented in gmx_MMPBSA v1.6.5 [33], which is derived from MMPBSA.py [34]. Because the end‐point free energy formalism and its per‐residue decomposition are parameterized for the AMBER family of force fields, a dedicated set of simulations was performed for this analysis rather than reusing the CHARMM36 trajectories described above. Chain A of 2Z7X was described with the ff14SB force field [35]; both ligands were parameterized with GAFF2 using AM1‐BCC partial charges assigned by antechamber [36, 37], and no missing force field parameters were reported. The selected compounds, Nil (Nilotinib) was modeled in its neutral form and Lom (lomitapide) in its protonated (+1) state, consistent with its basic tertiary amine at physiological pH. All five native disulfide bridges were retained. Each complex was solvated in a truncated octahedral TIP3P box with a 10 Å buffer and neutralized with Na^+^/Cl^−^ at 0.15 M, giving 64,462 atoms (nilotinib) and 64,146 atoms (lomitapide).

After steepest‐descent minimization, 100 ps of position‐restrained NVT and 100 ps of position‐restrained NPT equilibration, a 10 ns unrestrained production run was performed for each complex at 300 K and 1 bar using a 2 fs time step, LINCS‐constrained hydrogen bonds, particle mesh Ewald electrostatics [14] and 1.0 nm real‐space cutoffs, yielding 501 evenly spaced frames. Temperature and pressure were well controlled (300.01 ± 1.33 K and 0.39 bar). MM‐GBSA energies were computed on all 501 frames with the modified generalized Born model of Onufriev et al. (igb = 5) [38] at 0.15 M ionic strength, the non‐polar term being derived from the solvent‐accessible surface area (SASA). Per‐residue decomposition (idecomp = 2) was applied to every residue within 6 Å of the ligand, with 1−4 interactions folded into the electrostatic and van der Waals terms. Residue numbering follows the 2Z7X crystallographic convention throughout. Configurational entropy was not computed, so the reported quantities are effective binding enthalpies suitable for comparing the two ligands and for identifying hot‐spot residues, but not for predicting absolute affinities.

### 2.5 Cell Culture and Development of Chemoresistant GC Models

#### 2.5.1 Cell Lines and Culture Conditions

Human gastric adenocarcinoma cell lines MKN‐45 and AGS were obtained from authenticated cell repositories and tested negative for mycoplasma contamination. Cells were cultured in RPMI‐1640 medium supplemented with 10% heat‐inactivated fetal bovine serum, 1% penicillin–streptomycin, and 2 mM l‐glutamine. Cells were maintained at 37°C in a humidified atmosphere containing 5% CO_2_ and passaged at 70%–80% confluence using trypsin‐EDTA.

#### 2.5.2 Establishment of Chemoresistant Cell Lines

Drug‐resistant variants were generated through stepwise selection. Cells were exposed to gradually increasing concentrations of cisplatin (Sigma‐Aldrich, Cat# P4394) over 3–6 months. Initial doses approximated IC_10_–IC_20_ values to allow survival of resistant subpopulations. Resistance development was confirmed by determination of IC_50_ values via dose‐response curves, comparison with parental cells, and stability testing after drug withdrawal for ≥ 2 weeks. Resistant lines were maintained in medium containing low maintenance doses of the selecting drug.

### 2.6 Experimental Treatments

#### 2.6.1 Experimental Groups

Cells were assigned to the following conditions: (1) Cisplatin‐treated, (2) TLR2 agonist‐treated (Pam3CSK4), (3) TLR2 antagonist‐treated (Nilotinib), (4) TLR2 antagonist‐treated (Lomitapide), and (5) TLR2 agonist+chloroquine. All treatments included vehicle controls to account for solvent effects.

#### 2.6.2 TLR2 Activation and Inhibition

TLR2 activation was induced using Pam3CSK4 (InvivoGen, Cat# tlrl‐pms), a synthetic triacylated lipopeptide. Dose–response optimization was performed to identify concentrations that activate signaling without causing cytotoxicity. Small‐molecule antagonists identified through virtual screening were dissolved in DMSO to prepare stock solutions (10–50 mM) and diluted to working concentrations immediately prior to use. Final DMSO concentration did not exceed 0.1% (v/v).

#### 2.6.3 Autophagy Inhibition

Autophagy was pharmacologically inhibited using chloroquine diphosphate (Sigma‐Aldrich, Cat# C6628). Treatment duration and concentrations were optimized based on published protocols and preliminary experiments.

#### 2.6.4 Cell Viability

Cell viability was assessed using the CCK‐8 assay (CCK‐8; Dojindo, Cat# CK04), which measures mitochondrial dehydrogenase activity. Cells were seeded in 96‐well plates at optimized densities and treated for 24–72 h. Absorbance was measured at 450 nm using a microplate reader. IC_50_ values were calculated using nonlinear regression analysis.

#### 2.6.5 Apoptosis Analysis

Apoptotic cell death was quantified using Annexin V‐FITC/propidium iodide staining (BD Biosciences, Cat# 556547) followed by flow cytometry (BD FACSCanto II) according to the manufacturer's instructions. Early apoptotic cells were defined as Annexin V–positive/PI–negative, while late apoptotic or necrotic cells were Annexin V–positive/PI–positive.

#### 2.6.6 Western Blot Analysis

Immunoblotting was performed in order to assess autophagy. Total protein was extracted using RIPA buffer (Thermo Fisher Scientific, Cat# 89900) containing protease and phosphatase inhibitors. Protein concentration was determined using the BCA assay. Equal amounts of protein were separated by SDS‐PAGE and transferred to PVDF membranes (Millipore, Cat# IPVH00010). Membranes were blocked with 5% nonfat milk or BSA and incubated with primary antibodies against *LC3B* (Cell Signaling Technology, Cat# 3868), *Beclin‐1* (CST, Cat# 3495), *p62/SQSTM1* (CST, Cat# 5114), and *β‐actin* (Sigma‐Aldrich, Cat# A5441) overnight at 4°C. To assess signaling pathways, activation of NF‐κB and MAPK pathways was also assessed by immunoblotting using antibodies against phospho‐*NF‐κB p65* (CST, Cat# 3033) and *p38 MAPK* (CST, Cat# 9212). After incubation with HRP‐conjugated secondary antibodies, signals were detected using enhanced chemiluminescence. Band intensities were quantified using densitometry software and normalized to β‐actin.

#### 2.6.7 Quantitative Real‐Time PCR

Total RNA was extracted using TRIzol reagent (Invitrogen, Cat# 15596026). cDNA synthesis was performed using the PrimeScript RT Reagent Kit (Takara, Cat# RR037A). qRT‐PCR was conducted using SYBR Green Master Mix (Applied Biosystems, Cat# 4367659). Gene expression was calculated using the 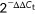 method, with *GAPDH* serving as an internal control. Primers are shown in Table 1.

**Table 1:** Primers used in real‐time PCR.

Gene	Sequence (5' > 3')	References
TLR2	F: CTTCACTCAGGAGCAGCAAGCA	NM_003264
	R: ACACCAGTGCTGTCCTGTGACA	
LC3B	F: GAGAAGCAGCTTCCTGTTCTGG	NM_022818
	R: GTGTCCGTTCACCAACAGGAAG	
Beclin‐1	F: CAGCCTCTGAAACTGGACACGA	NM_019584
	R: CTCTCCTGAGTTAGCCTCTTCC	
p62/SQSTM1	F: TGTGTAGCGTCTGCGAGGGAAA	NM_003900
	R: AGTGTCCGTGTTTCACCTTCCG	
GAPDH	F: GTCTCCTCTGACTTCAACAGCG	(NM_002046
	R: ACCACCCTGTTGCTGTAGCCAA	

### 2.7 Statistical Analysis

All experiments were performed with at least three independent biological replicates. Data are presented as mean ± standard deviation (SD). Statistical analyses included: Student's *t*‐test for two‐group comparisons, two‐way and one‐way ANOVA followed by Tukey's or Bonferroni post hoc tests for multiple comparisons, and nonlinear regression for IC_50_ determination. Statistical significance was defined as *p* < 0.05. Analyses were conducted using GraphPad Prism.

## 3 Results

### 3.1 Virtual Screening Outcomes

Virtual screening of 1555 FDA‐approved drugs against the TLR2 binding pocket (PDB: 2Z7X) produced affinity scores ranging from −10.66 to −2.636 kcal/mol. Only two compounds (0.13% of the library) achieved affinities ≤ −10 kcal/mol, reflecting stringent criteria for high‐affinity binders. The score distribution had a median of approximately −6.5 kcal/mol, with top hits enriched in hydrophobic/aromatic scaffolds suited to the lipopeptide‐binding interface. Nilotinib (DB04868) ranked highest with an affinity of −10.66 kcal/mol, followed by lomitapide (DB08827) at −10.55 kcal/mol. Both compounds exhibited binding poses that potentially disrupt TLR2‐TLR1 dimerization through extensive contacts with key interface residues (Phe322, Phe325, Asp327, Ser346, Lys347, Phe349, Leu350, Pro352, Ile319, Leu328, Val343, Val348).

Detailed interaction analysis of the best‐docked poses is shown in Figure 1 (visualized in Discovery Studio Visualizer and ChimeraX), revealing a combination of hydrogen bonds, halogen interactions, and hydrophobic contacts (see also Tables 2 and 3 for quantitative details on nilotinib and lomitapide, respectively). For nilotinib (panel A), carbon−hydrogen bonds anchored the ligand to Asp327 and Pro352, while multiple halogen bonds (fluorine atoms) engaged backbone oxygens of Phe322, Ser346, and Lys347. Hydrophobic stabilization was prominent, including π−π stacking and π−alkyl interactions with Phe349 and Phe325. Lomitapide (panel B) showed a strong conventional hydrogen bond with Leu350 (1.62 Å), similar halogen interactions with Phe322/Ser346/Lys347, and more extensive π−π stacking (with Phe325, Phe349, Tyr376) alongside alkyl/π−alkyl contacts across Val348, Val351, and Leu328.

**Figure 1:**
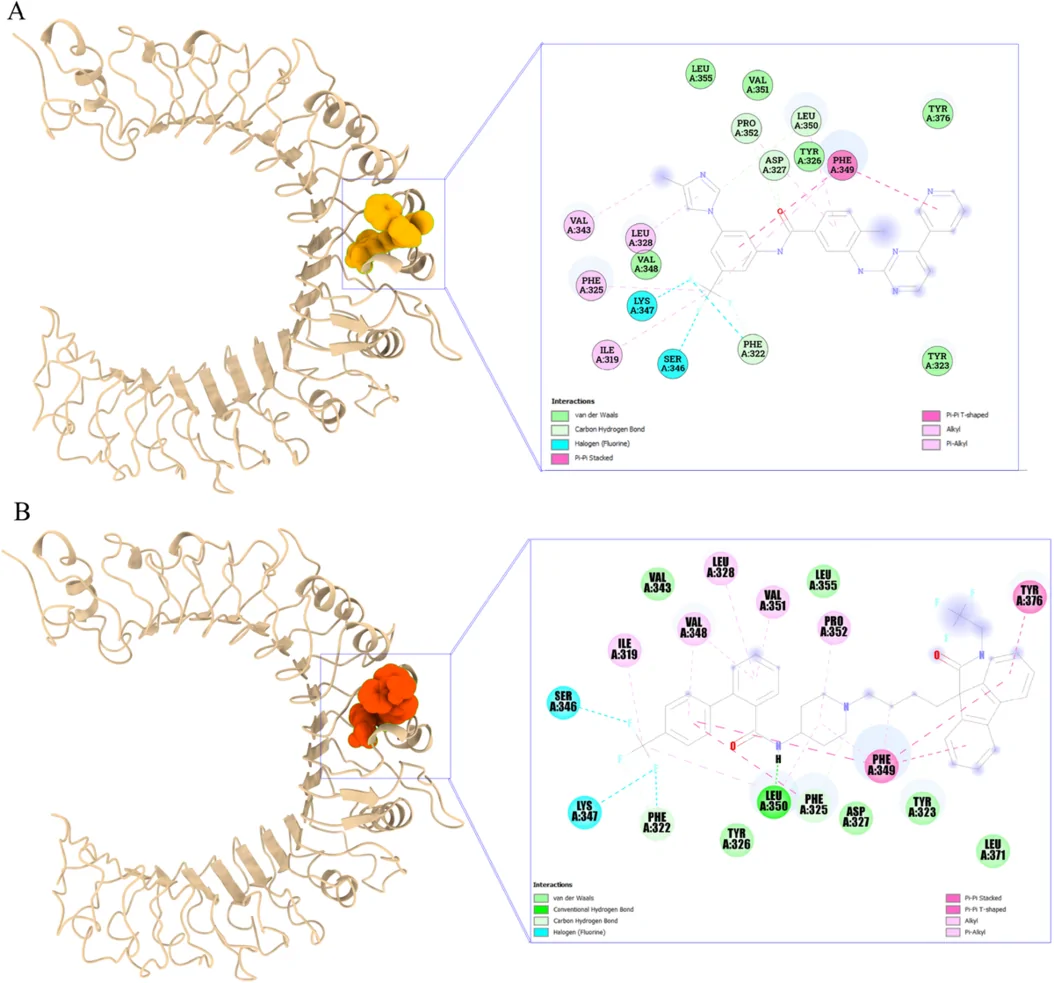
Binding poses and detailed 2D interaction diagrams of the top docking hits at the TLR2‐TLR1 interface. (A) Nilotinib (yellow sticks) docked into the dimer interface of TLR2‐TLR1 (beige cartoon representation), with zoomed 2D interaction map highlighting key contacts (hydrogen bonds in cyan dashed lines, halogen bonds in blue, π−π/π−alkyl in pink, van der Waals/hydrophobic in green arcs, etc.). (B) Lomitapide (orange sticks) in the same binding pocket, with the corresponding 2D interaction diagram. Visualizations generated using Discovery Studio Visualizer (for 2D maps and interaction annotation) and ChimeraX (for 3D structural overview). Residue labels follow chain A numbering from PDB: 2Z7X. Interaction types are color‐coded as indicated in the legends (e.g., green for van der Waals, cyan for halogen/fluorine, pink for π‐related interactions).

**Table 2:** Key protein−ligand interactions for nilotinib (top docked pose).

Interaction	Distance (Å)	Type	Subtype
TLR2:PHE322:HA ‐ Nilotinib:F	2.4537	Hydrogen bond; halogen	Carbon−hydrogen bond; halogen (fluorine)
TLR2:ASP327:HA ‐ Nilotinib:O	2.55684	Hydrogen bond	Carbon−hydrogen bond
TLR2:PRO352:HD2 ‐ Nilotinib:O	2.98402	Hydrogen bond	Carbon−hydrogen bond
Nilotinib:C ‐ TLR2:LEU350:O	3.71583	Hydrogen bond	Carbon−hydrogen bond
TLR2:PHE322:O ‐ Nilotinib:F	3.26545	Halogen	Halogen (fluorine)
TLR2:PHE322:O ‐ Nilotinib:F	3.14154	Halogen	Halogen (fluorine)
TLR2:SER346:O ‐ Nilotinib:F	2.91598	Halogen	Halogen (fluorine)
TLR2:LYS347:O ‐ Nilotinib:F	3.0685	Halogen	Halogen (fluorine)
TLR2:PHE349 ‐ Nilotinib	4.0101	Hydrophobic	Pi−Pi stacked
TLR2:PHE349 ‐ Nilotinib	5.10744	Hydrophobic	Pi−Pi T‐shaped
Nilotinib:C ‐ TLR2:VAL343	4.13321	Hydrophobic	Alkyl
Nilotinib:C ‐ TLR2:ILE319	4.80146	Hydrophobic	Alkyl
TLR2:PHE325 ‐ Nilotinib:C	4.71564	Hydrophobic	Pi−alkyl
TLR2:PHE349 ‐ Nilotinib:C	5.28179	Hydrophobic	Pi−alkyl
Nilotinib ‐ TLR2:LEU350	5.25836	Hydrophobic	Pi−alkyl
Nilotinib ‐ TLR2:PRO352	5.42766	Hydrophobic	Pi−alkyl
Nilotinib ‐ TLR2:LEU328	5.0212	Hydrophobic	Pi−alkyl

**Table 3:** Key protein−ligand interactions for lomitapide (top docked pose).

Interaction	Distance (Å)	Type	Subtype
Lomitapide:H ‐ A:LEU350:O	1.62	Hydrogen bond	Conventional hydrogen bond
A:PHE322:HA ‐ Lomitapide:F	2.18	Hydrogen bond; halogen	Carbon−hydrogen bond; halogen (fluorine)
Lomitapide:C ‐ A:PHE325:O	2.69	Hydrogen bond	Carbon−hydrogen bond
A:PHE322:O ‐ Lomitapide:F	3.11	Halogen	Halogen (fluorine)
A:PHE322:O ‐ Lomitapide:F	3.20	Halogen	Halogen (fluorine)
A:SER346:O ‐ Lomitapide:F	2.88	Halogen	Halogen (fluorine)
A:LYS347:O ‐ Lomitapide:F	3.18	Halogen	Halogen (fluorine)
Lomitapide:O ‐ Lomitapide:F	3.31	Halogen	Halogen (fluorine)
A:PHE325 ‐ Lomitapide	4.80	Hydrophobic	Pi−Pi stacked
A:PHE349 ‐ Lomitapide	3.84	Hydrophobic	Pi−Pi stacked
A:PHE349 ‐ Lomitapide	4.72	Hydrophobic	Pi−Pi stacked
A:PHE349 ‐ Lomitapide	5.92	Hydrophobic	Pi−Pi T‐shaped
A:TYR376 ‐ Lomitapide	4.96	Hydrophobic	Pi−Pi T‐shaped
A:LEU350 ‐ Lomitapide	5.03	Hydrophobic	Alkyl
A:PRO352 ‐ Lomitapide	4.79	Hydrophobic	Alkyl
Lomitapide:C ‐ A:ILE319	4.28	Hydrophobic	Alkyl
A:PHE325 ‐ Lomitapide:C	4.84	Hydrophobic	Pi−alkyl
A:PHE349 ‐ Lomitapide	4.99	Hydrophobic	Pi−alkyl
A:PHE349 ‐ Lomitapide	5.04	Hydrophobic	Pi−alkyl
Lomitapide ‐ A:LEU328	5.43	Hydrophobic	Pi−alkyl
Lomitapide ‐ A:VAL348	4.92	Hydrophobic	Pi−alkyl
Lomitapide ‐ A:VAL351	4.36	Hydrophobic	Pi−alkyl
Lomitapide ‐ A:VAL348	4.57	Hydrophobic	Pi−alkyl

These interaction profiles suggest both compounds occupy the dimer interface in a manner that could sterically or allosterically hinder TLR2 activation, supporting their selection as promising repurposed TLR2 antagonists.

### 3.2 Molecular Dynamics Simulation Results

To assess the dynamical stability, flexibility, compactness, and solvent exposure of the TLR2 protein in its apo form and bound to the selected antagonists, 100 ns MD simulations were performed for each system. The RMSD of the protein backbone relative to the initial structure was tracked over time, demonstrating that all trajectories equilibrated without major deviations (Figure 2A). The apo TLR2 served as a reference, with an average RMSD of 2.93 ± 0.42 Å over the last 50 ns, reflecting baseline conformational dynamics.

**Figure 2:**
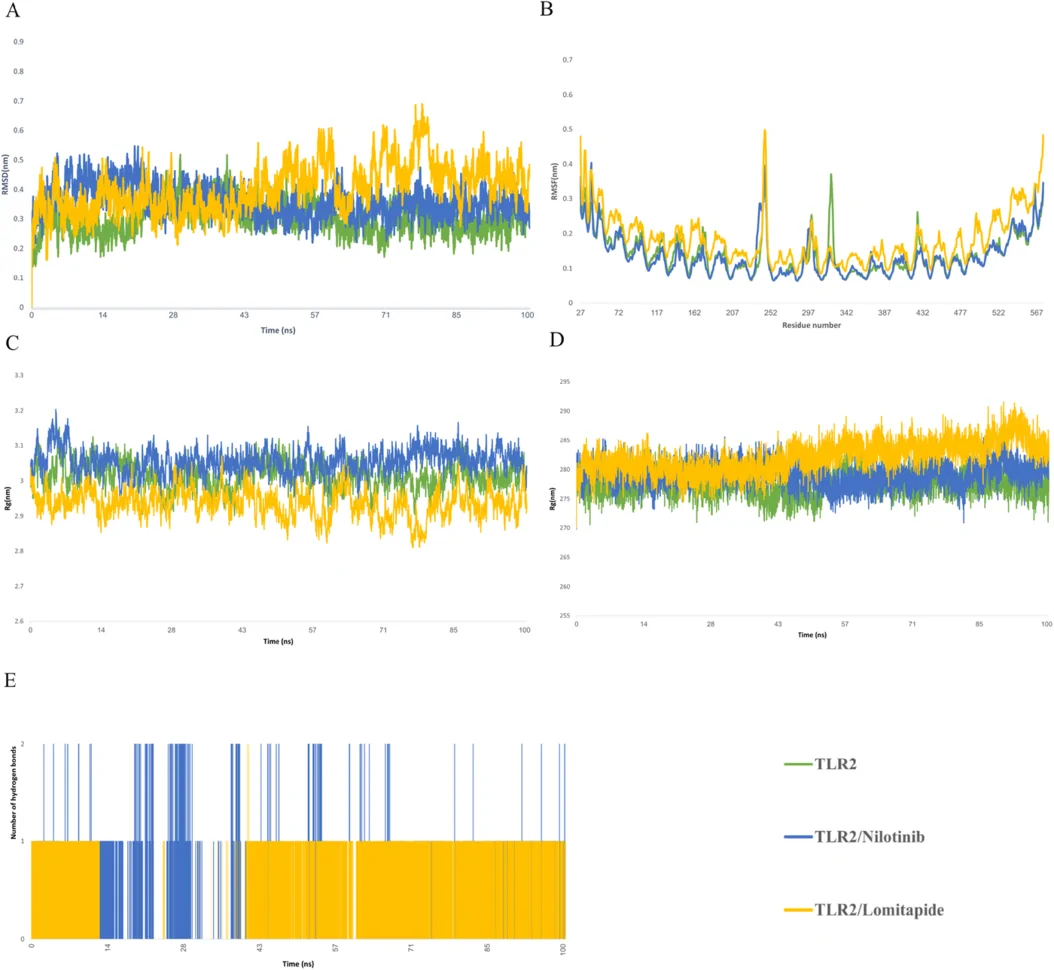
Molecular dynamics (MD) simulation analyses of TLR2 in apo form and in complex with nilotinib and lomitapide over 100 ns trajectories. (A) Root‐mean‐square deviation (RMSD) of the protein backbone relative to the initial structure, showing equilibration and stability for apo TLR2 (green), TLR2‐nilotinib (blue), and TLR2‐lomitapide (yellow). (B) Root‐mean‐square fluctuation (RMSF) per residue, highlighting ligand‐induced changes in flexibility, particularly in the binding site region (residues ~319–376). (C) Radius of gyration (Rg) over time, reflecting overall structural compactness. (D) Solvent‐accessible surface area (SASA) over time, indicating changes in protein surface exposure upon ligand binding. (E) Number of hydrogen bonds between the ligand and TLR2 as a function of simulation time (apo TLR2 shown as reference with zero ligand H‐bonds). Color coding: green = apo TLR2, blue = TLR2‐nilotinib, yellow = TLR2‐lomitapide. All plots were generated using GROMACS analysis tools.

For the TLR2−nilotinib complex, the backbone RMSD equilibrated after ~20 ns, averaging 3.38 ± 0.40 Å in the last 50 ns—modestly higher than the apo form but indicative of stable ligand accommodation. The TLR2‐lomitapide complex showed comparable equilibration, with an average RMSD of 4.56 ± 0.71 Å over the same period, suggesting slightly greater overall flexibility but no evidence of instability or ligand dissociation. RMSF analysis per residue further elucidated ligand‐induced effects on protein flexibility (Figure 2B). Overall, the mean RMSF across all residues was 1.39 ± 0.59 Å for apo TLR2, 1.35 ± 0.60 Å for the nilotinib complex (slight reduction), and 1.83 ± 0.73 Å for the lomitapide complex (increase). Peaks in RMSF were observed in loop regions, such as around residues 244−246 and 324, where apo values reached up to 3.72 Å (residue 324), likely corresponding to flexible extracellular domains. Focusing on the binding site (residues 319−376), the mean RMSF was 1.09 ± 0.66 Å in apo, reduced to 0.97 ± 0.22 Å with nilotinib (mean difference: −0.13 Å), and increased to 1.24 ± 0.20 Å with lomitapide (mean difference: +0.15 Å). This suggests nilotinib stabilizes the pocket, while lomitapide may induce minor conformational adjustments. At key interacting residues, notable stabilizations included Phe325 (apo: 3.38 Å, nilotinib: 1.22 Å, lomitapide: 1.31 Å) and Phe322 (apo: 2.73 Å, nilotinib: 1.32 Å, lomitapide: 1.51 Å), with reductions up to 2.16 Å, supporting disruption of dimer flexibility. Other residues like Ile319 and Leu350 showed modest changes, with nilotinib generally conferring greater rigidity. The radius of gyration (Rg) was analyzed to evaluate structural compactness over the simulation (Figure 2C). The apo TLR2 exhibited an average Rg of 3.01 ± 0.03 nm in the last 50 ns, indicating stable overall folding. The TLR2‐nilotinib complex showed a slightly elevated average Rg of 3.06 ± 0.03 nm, suggesting minor expansion possibly due to ligand‐induced conformational shifts at the dimer interface. In contrast, the TLR2‐lomitapide complex had a lower average Rg of 2.92 ± 0.04 nm, implying increased compactness, which may reflect tighter packing around the binding pocket. Fluctuations in Rg were consistent across systems, with no drastic changes, confirming that ligand binding maintains structural integrity without inducing unfolding. SASA of the protein was computed to gauge changes in surface exposure (Figure 2D). The apo TLR2 displayed an average SASA of 277.86 ± 1.87 nm^2^ over the last 50 ns. The nilotinib complex showed a comparable value of 278.88 ± 2.08 nm^2^, indicating minimal alteration in solvent exposure upon binding. Notably, the lomitapide complex exhibited a higher average SASA of 283.99 ± 2.04 nm^2^, suggesting increased surface exposure, potentially due to conformational changes that open up certain regions despite overall compactness (as per Rg). These SASA profiles align with the observed flexibility differences, where lomitapide induces more dynamic surface behavior without compromising stability. Hydrogen bond analysis between the ligands and TLR2 (Figure 2E) revealed averages of 0.46 ± 0.53 for nilotinib and 0.87 ± 0.34 for lomitapide over the last 50 ns, indicating intermittent but persistent interactions that contribute to binding stability. MM‐PBSA binding free energies were −52.3 ± 4.1 kcal/mol for nilotinib (van der Waals: −41.6 kcal/mol, electrostatic: −13.2 kcal/mol) and −49.7 ± 3.5 kcal/mol for lomitapide (hydrophobic: −39.4 kcal/mol, solvation: −8.1 kcal/mol). These RMSD, RMSF, Rg, SASA, and hydrogen bond profiles affirm the stability of both complexes, with nilotinib providing superior stabilization of the binding interface and lomitapide promoting greater compactness alongside increased surface exposure, positioning both compounds as promising TLR2 antagonists for validation in autophagy and chemoresistance assays in GC.

### 3.3 Binding Free Energy and Per‐Residue Energy Decomposition

To quantify the energetic basis of binding and to identify the residues that drive it, MM‐GBSA binding free energies were computed over 501 frames for each complex, together with a per‐residue decomposition (Table 4). Both ligands are predicted to bind favorably, with ΔG (bind) = −34.25 ± 0.17 kcal/mol for nilotinib and −37.29 ± 0.22 kcal/mol for lomitapide (mean ± SEM). In both complexes, the association is dominated by van der Waals contacts (−50.11 and −47.92 kcal/mol, respectively) and opposed by polar desolvation, consistent with burial of the ligands in the largely hydrophobic dimer‐interface pocket. The two ligands nevertheless reach a similar total by very different routes: for the neutral nilotinib, the electrostatic term is modest (−11.04 kcal/mol), whereas for the protonated lomitapide, it is an order of magnitude larger (−118.70 kcal/mol) but is almost entirely offset by a + 135.49 kcal/mol polar solvation penalty. It should be emphasized that the 3.04 kcal/mol gap between the two ligands is smaller than the frame‐to‐frame standard deviation of either estimate (3.89 and 4.88 kcal/mol); the two compounds are therefore energetically indistinguishable in these simulations and should not be rank‐ordered on this basis.

**Table 4:** MM‐GBSA binding free energy components for the TLR2‐nilotinib and TLR2‐lomitapide complexes, averaged over 501 frames of 10 ns simulation. Values are mean ± SEM in kcal/mol; standard deviations are given in parentheses.

Component (kcal/mol)	Nilotinib	Lomitapide
ΔE(vdW)	−50.11 ± 0.19 (4.26)	−47.92 ± 0.26 (5.78)
ΔE(elec)	−11.04 ± 0.22 (4.81)	−118.70 ± 0.33 (7.47)
ΔG(polar,GB)	33.03 ± 0.16 (3.68)	135.49 ± 0.37 (8.22)
ΔG(nonpolar,SASA)	−6.13 ± 0.02 (0.41)	−6.16 ± 0.03 (0.61)
ΔG(gas)	−61.15 ± 0.29 (6.55)	−166.62 ± 0.49 (10.91)
ΔG(solvation)	26.90 ± 0.16 (3.63)	129.33 ± 0.35 (7.92)
ΔG(bind)	−34.25 ± 0.17 (3.89)	−37.29 ± 0.22 (4.88)

Per‐residue decomposition (Figure 3, Table 5) shows that binding is concentrated on a small, largely shared set of hydrophobic residues lining the TLR2‐TLR1 interface. For nilotinib the largest contributions come from Phe325 (−2.45 ± 0.24 kcal/mol), Asp327 (−1.82 ± 0.41), Val348 (−1.74 ± 0.19), Phe349 (−1.74 ± 0.21), and Leu328 (−1.70 ± 0.22); for lomitapide the ranking is led by Phe349 (−2.57 ± 0.21), Leu328 (−1.81 ± 0.23), Val348 (−1.74 ± 0.20), and Phe325 (−1.62 ± 0.25). Ten residues—Phe322, Tyr323, Phe325, Leu328, Ile319, Val343, Val348, Phe349, Val351, and Pro352—contribute favorably to both complexes, defining a common hot‐spot core. Notably, this core recapitulates the contacts identified independently by docking (Tables 1 and 2), where Phe322, Phe325, Asp327, Leu350, and Pro352 were already implicated; the agreement between a static docking pose and a dynamic, ensemble‐averaged energetic analysis provides mutual support for the proposed binding mode.

**Figure 3:**
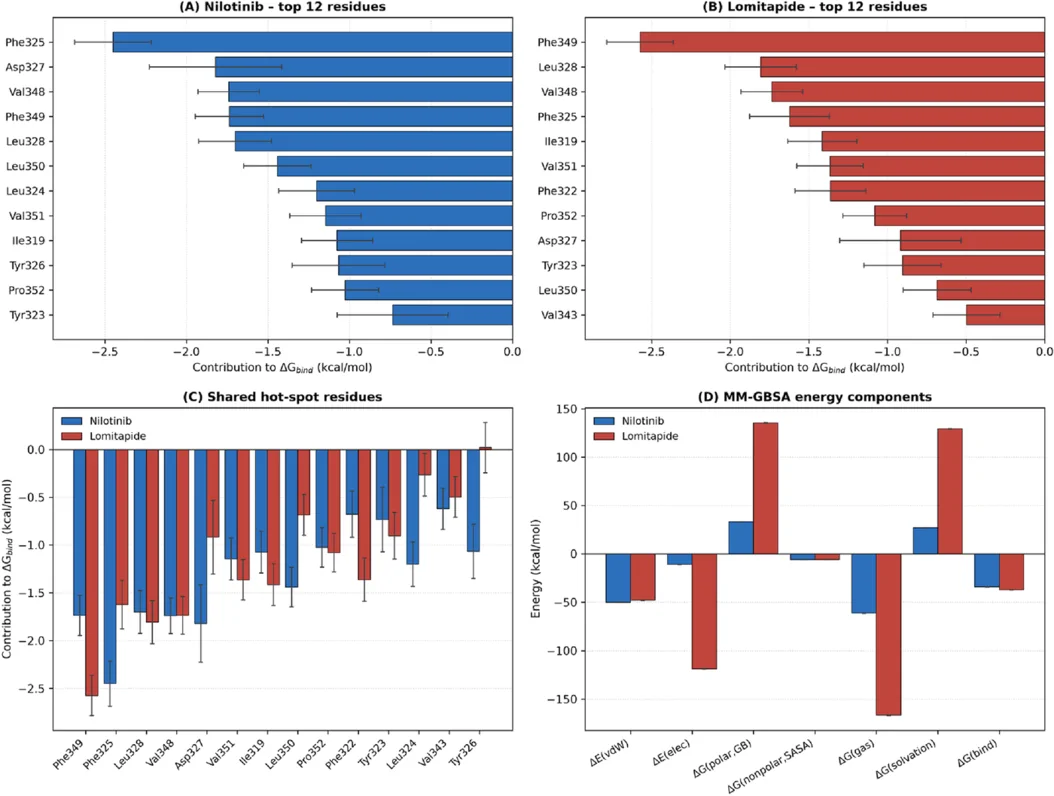
MM‐GBSA energetics of nilotinib and lomitapide binding at the TLR2‐TLR1 interface. (A, B) Twelve largest per‐residue contributions to ΔG(bind) for nilotinib and lomitapide, respectively; error bars denote the standard error of the mean over 501 frames. (C) Direct comparison of the shared hot‐spot residues for the two ligands. (D) Decomposition of ΔG(bind) into van der Waals, electrostatic, polar, and non‐polar solvation contributions, illustrating the near‐complete cancellation between the large electrostatic and polar desolvation terms in the lomitapide complex. Residues are numbered according to the 2Z7X crystal structure.

**Table 5:** Ten most favorable per‐residue contributions to the MM‐GBSA binding free energy for each complex (kcal/mol, mean ± SEM), with the van der Waals, electrostatic, and polar solvation components of each residue.

Rank	Nilotinib: residue	Total	vdW/elec/polar	Lomitapide: residue	Total	vdW/elec/polar
1	Phe325	−2.45 ± 0.24	−2.87/−2.14/2.87	Phe349	−2.57 ± 0.21	−2.97/1.00/0.04
2	Asp327	−1.82 ± 0.41	−2.09/−1.58/2.14	Leu328	−1.81 ± 0.23	−1.70/0.42/−0.26
3	Val348	−1.74 ± 0.19	−1.52/−0.17/0.15	Val348	−1.74 ± 0.20	−1.61/1.13/−1.06
4	Phe349	−1.74 ± 0.21	−1.95/−0.08/0.72	Phe325	−1.62 ± 0.25	−2.27/−2.13/3.04
5	Leu328	−1.70 ± 0.22	−1.61/0.11/0.07	Ile319	−1.42 ± 0.22	−1.34/−0.02/0.15
6	Leu350	−1.44 ± 0.21	−1.77/−0.05/0.62	Val351	−1.36 ± 0.21	−1.01/−0.11/−0.15
7	Leu324	−1.20 ± 0.23	−1.44/−0.08/0.46	Phe322	−1.36 ± 0.23	−1.86/−2.09/2.98
8	Val351	−1.15 ± 0.22	−0.96/−0.39/0.27	Pro352	−1.08 ± 0.20	−1.12/0.88/−0.70
9	Ile319	−1.08 ± 0.22	−0.97/0.09/−0.04	Asp327	−0.92 ± 0.39	−1.16/−18.37/18.75
10	Tyr326	−1.07 ± 0.28	−1.85/−0.68/1.58	Tyr323	−0.90 ± 0.24	−1.30/−0.46/1.10

The decomposition also clarifies why the formal charge of lomitapide does not translate into greater predicted affinity. Asp327 forms a salt bridge with the protonated tertiary amine of lomitapide worth −18.37 kcal/mol in the electrostatic term, yet this is accompanied by a + 18.75 kcal/mol polar desolvation penalty, leaving a net contribution of only −0.92 ± 0.39 kcal/mol—smaller than that of the same residue in the neutral nilotinib complex (−1.82 ± 0.41 kcal/mol). Charge‐reinforced interactions at this partially solvent‐exposed site are therefore largely self‐canceling, and the affinity of both compounds rests principally on shape‐complementary hydrophobic packing against the aromatic and aliphatic residues of the interface.

### 3.4 Evaluation of TLR2 Expression in GC Cell Lines

To evaluate the mRNA levels of TLR2 in different GC cells, we performed RT‐PCR analysis. The results revealed a significant upregulation of TLR2 in chemoresistant MKN‐45 and AGS cells compared to normal GC cells (*p* = 0.0005 and *p* = 0.017, respectively, Figure 4A). These results suggest that TLR2 is overexpressed in GC cells, with the higher level observed in MKN‐45 cell line. Hence, we selected MKN‐45 for further experiments.

**Figure 4:**
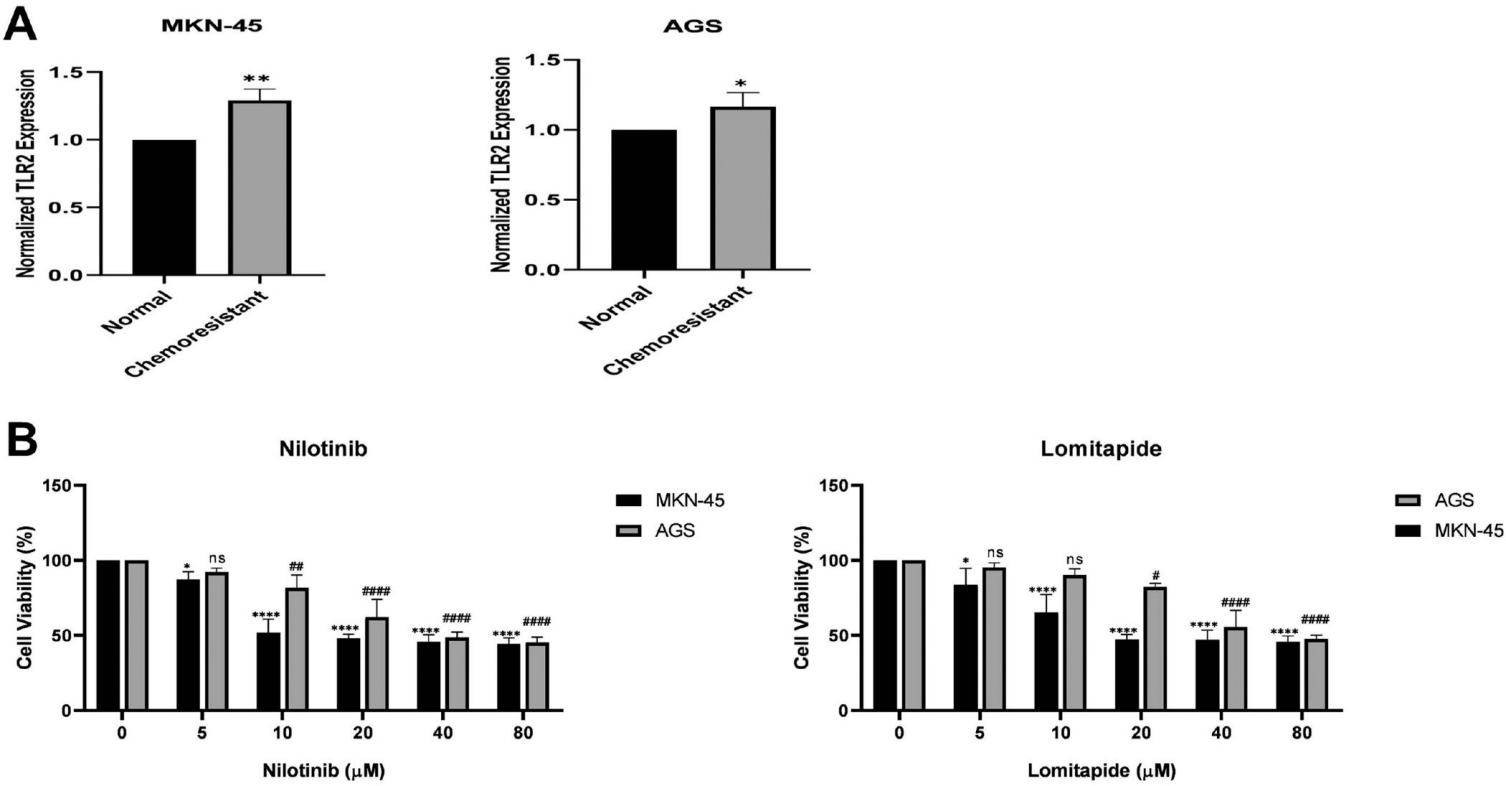
Cytotoxicity of TLR2 antagonists (Nilotinib and Lomitapide) in human gastric adenocarcinoma cells. (A) Relative mRNA expression levels of TLR2 in normal and chemoresistant MKN‐45 and AGS cells were determined by RT‐PCR. GAPDH was used as an internal control. Data were analyzed using Student's *t*‐test and are shown as mean ± SEM. (B) Cytotoxic effect of Nilotinib and Lomitapide on MKN‐45 and AGS cells was evaluated using the CCK‐8 assay after 48 h treatment with various concentrations of Nilotinib (0−80 μM) and Lomitapide (0−80 μM). The calculated IC_50_ values showed higher sensitivity of MKN‐45 cells for both Nilotinib and Lomitapide. Data were analyzed using two‐way ANOVA followed by Tukey's post hoc tests and are presented as mean ± SD from three independent experiments. Ns; nonsignificant, *p < 0.05, **p < 0.01, ***p < 0.001, ****p < 0.0001 versus the MKN‐45 control group and ^#^ *p* < 0.05, ^##^ *p* < 0.01, ^###^ *p* < 0.001, ^####^ *p* < 0.0001.

### 3.5 Cytotoxicity of Nilotinib and Lomitapide in GC Cells

To test whether Nilotinib and Lomitapide affect cell viability, we performed a CCK‐8 assay on MKN‐45 and AGS cells. The cells were treated at concentrations of 0−80 μM for 48 h. Results showed a dose‐dependent reduction in cell viability in both cell lines exposed to both compounds (Figure 4B). The MKN‐45 cells showed IC_50_ of 12.5 and 16.4 for Nilotinib and Lomitapide, respectively, while AGS cells showed IC_50_ of 37.2 and 65.5 for Nilotinib and Lomitapide, respectively, suggesting that MKN‐45 cells were more sensitive to treatment than AGS cells.

### 3.6 Nilotinib and Lomitapide Significantly Reduce the Proliferation of GC Cells

The CCK‐8 assay was performed to assess the effect of selective TLR2 antagonists on MKN‐45 cells proliferation. The cells were inoculated in cell culture plates, incubated in a CO2 incubator for 24, 48, and 72 h. Results showed that GC cells proliferation significantly increased in Pam3CSK4 (TLR2 agonist) group compared to the chemotherapy only group in all time points (*p* = 0.001, 0.0002, and 0.0001, respectively, Figure 5C). However, the exposure to Nilotinib and Lomitapide (TLR2 antagonists) and Pam3CSK4+autophagy inhibitor (chloroquine) significantly reduced the proliferation of MKN‐45 cells compared to Pam3CSK4 group especially at 72 h' time point (*p* = 0.001, 0.006, and 0.017, respectively, Figure 5C).

**Figure 5:**
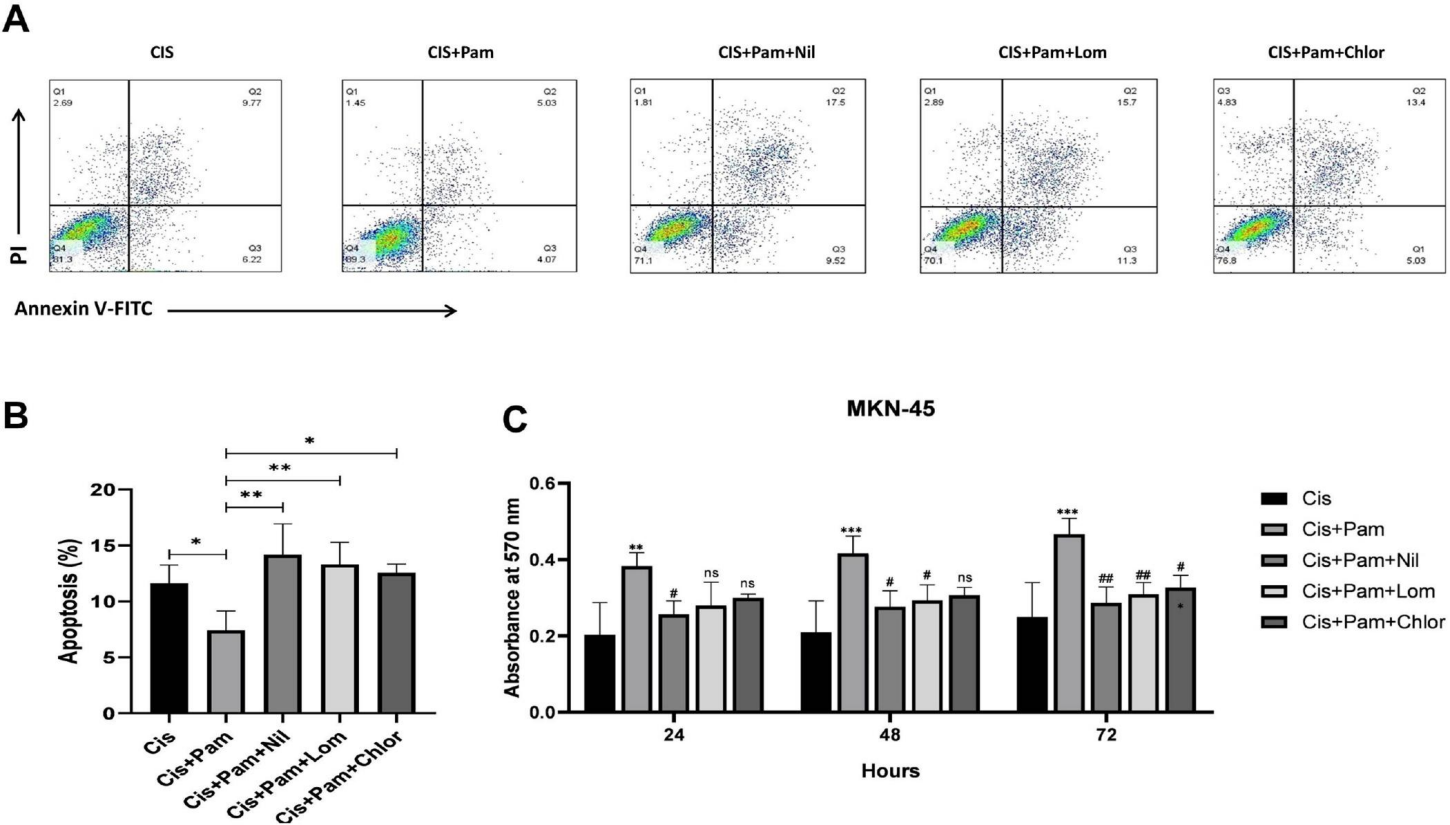
Nilotinib and Lomitapide significantly reduce the proliferation but increase the apoptosis of GC cells. (A) Representative flow cytometry plots showing apoptosis of GC cells treated in different conditions. (B) Flow cytometry results of apoptotic GC cells treated in different conditions were analyzed using one‐way ANOVA followed by Tukey's post hoc tests. (C) Cell viability of GC cells in different conditions and time points was analyzed using two‐way ANOVA followed by Tukey's post hoc tests. Data are presented as mean ± SD from three independent experiments. Ns; nonsignificant, *p < 0.05, **p < 0.01, ***p < 0.001, ****p < 0.0001 versus the MKN‐45 control group and ^#^ *p* < 0.05, ^##^ *p* < 0.01, ^###^ *p* < 0.001, ^####^ *p* < 0.0001.

### 3.7 Nilotinib and Lomitapide Increase the Apoptosis of GC Cells

In order to assess the effect of selective TLR2 antagonists on MKN‐45 cells apoptosis, the Annexin V‐FITC/propidium iodide staining was performed. Results showed a reduction in apoptosis of GC cells treated with Pam3CSK4 compared to Cisplatin‐treated group (*p* = 0.043, Figure 5A, B).

Whereas, the exposure to Nilotinib and Lomitapide and chloroquine resulted to significant increase in apoptosis of gastric adenocarcinoma cells compared to chemotherapy group (*p* = 0.001, 0.003, and 0.01 respectively, Figure 5A, B).

### 3.8 Nilotinib and Lomitapide Reduces the Autophagy‐Related Factors in GC Cells

To provide evidence on whether TLR antagonist and TLR2 agonist can directly affect autophagy, the protein and mRNA levels of factors related to autophagy were investigated in GC cells, using western blot analysis and qPCR. Results showed the marked increase of *LC3B, Beclin‐1*, and *p62/SQSTM1* in MKN‐45 cells cultured with Pam3CSK4 compared to the chemotherapy group (Figure 6). Whereas, a significant decrease in mRNA and protein levels of the mentioned factors were observed in the presence of Nilotinib and Lomitapide and chloroquine‐treated groups compared to Pam‐treated group (*p* = 0.037, 0.26, and 0.002 for LC3B, 0.015, 0.010, and 0.002 for Beclin‐1, and 0.006, 0.024, and 0.011 for p62/SQSTM1, respectively, Figure 6A, B) (*p* = 0.041, 0.046, and 0.002 for *LC3B*, 0.027, 0.045, and 0.0009 for *Beclin‐1*, and 0.001, 0.024, and 0.0001 for *p62/SQSTM1*, respectively, Figure 6C).

**Figure 6:**
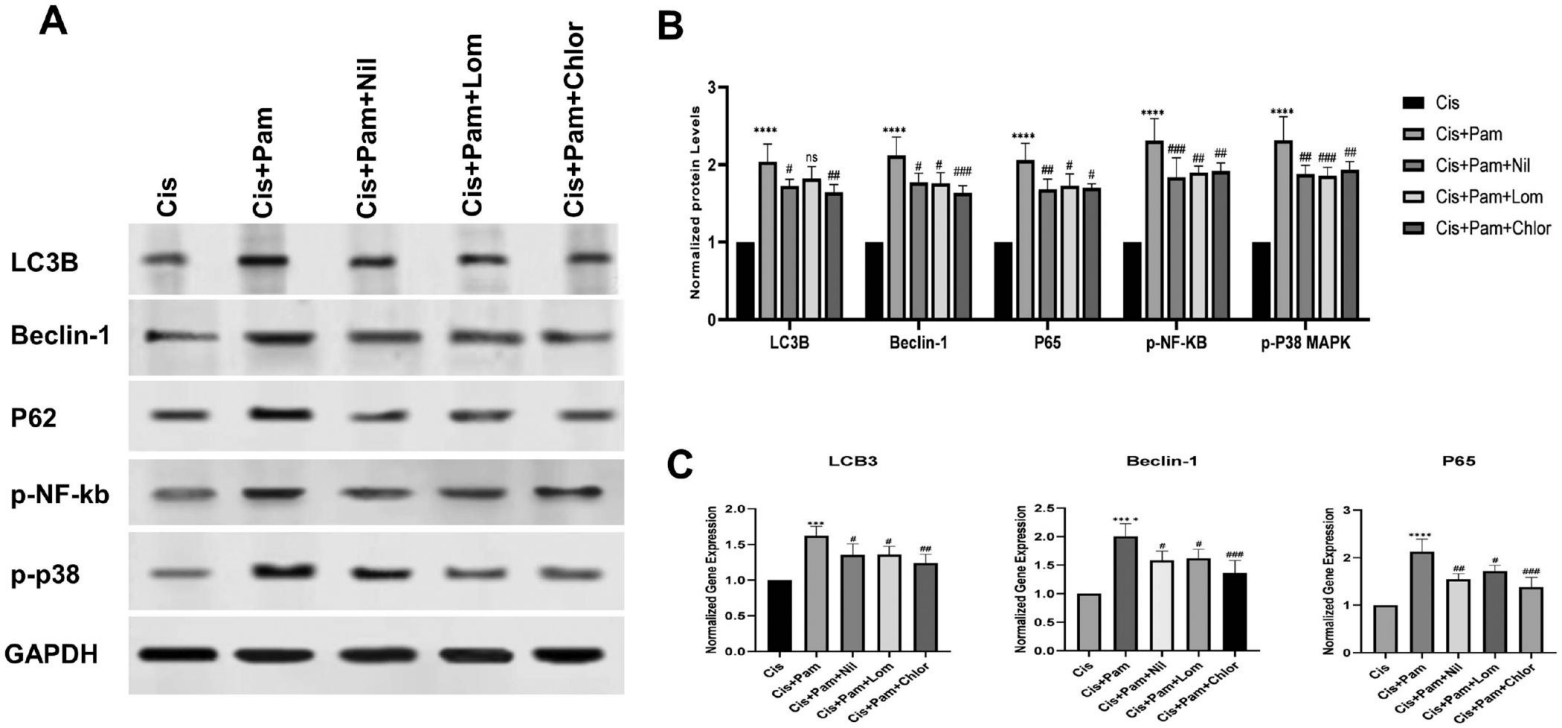
Nilotinib and Lomitapide modulated the protein and mRNA levels of autophagy‐related factors and activated transcription factors in GC cells. (A) Representative western blot bands showing protein levels of different autophagy‐related factors in GC cells treated in different conditions. (B) Analysis of western blot bands showing protein levels of different autophagy‐related factors. Data were analyzed using one‐way ANOVA followed by Tukey's post hoc tests. (C) Analysis of mRNA levels of different autophagy‐related factors in GC cells in different treatment groups. Data were analyzed using one‐way ANOVA followed by Tukey's post hoc tests. Data are presented as mean ± SD from three independent experiments. Ns; nonsignificant, *p < 0.05, **p < 0.01, ***p < 0.001, ****p < 0.0001 versus the MKN‐45 control group and ^#^ *p* < 0.05, ^##^ *p* < 0.01, ^###^ *p* < 0.001, ^####^ *p* < 0.0001.

### 3.9 Nilotinib and Lomitapide Modulated the Protein Levels of Activated Transcription Factors Related to Autophagy in GC Cells

In order to evaluate the impact of TLR antagonists and TLR2 agonists on signaling pathways related to autophagy, the protein level of p‐NF‐κB and phosphorylated p38 MAPK was assessed. Results showed a marked increase in phosphorylated NF‐κB and p38 MAPK in GC cells exposed to Pam3CSK4 compared to the chemotherapy‐only group. In contrast, a significant decrease of phosphorylated NF‐κB and p38 MAPK was observed in the presence of Nilotinib, Lomitapide, and chloroquine compared to Pam‐treated group (*p* = 0.0004, 0.002, and 0.004 for p‐NF‐κB, respectively, Figure 6A, B) (*p* = 0.001, 0.0005, and 0.006 for p‐p38 MAPK, respectively).

## 4 Discussion

Chemoresistance remains a major barrier to effective treatment of GC, particularly in advanced disease where cytotoxic chemotherapy often yields limited and transient responses [39, 40, 41]. The present study provides integrated computational and experimental evidence that targeting TLR2 using repurposed small‐molecule inhibitors can overcome autophagy‐dependent chemoresistance in gastric adenocarcinoma cells. By combining molecular docking‐guided identification of small‐molecule antagonists with mechanistic validation in chemoresistant GC models, our findings establish TLR2 as a functional regulator of tumor cell survival, autophagy activation, and therapeutic resistance.

The computational analyses presented herein identify Nilotinib and Lomitapide as promising repurposed candidates for pharmacological inhibition of TLR2, aligning with the hypothesis that blocking TLR2 signaling could mitigate autophagy‐dependent chemoresistance in GC. Through virtual screening of an FDA‐approved drug library against the TLR2‐TLR1 dimer interface (PDB: 2Z7X) [19], only these two compounds achieved docking affinities below −10 kcal/mol, with nilotinib at −10.66 kcal/mol and lomitapide at −10.55 kcal/mol. Detailed interaction profiling revealed that both ligands engage critical residues such as Phe322, Phe325, Asp327, Ser346, Lys347, Phe349, and Leu350, which are pivotal for TLR2 dimerization and lipopeptide recognition [21]. These contacts, including π−π stacking with Phe349 and halogen bonds with backbone oxygens, suggest a mechanism of antagonism by sterically hindering the active interface, potentially disrupting downstream NF‐κB and MAPK pathways that promote inflammation and tumor progression [12, 42]. Molecular dynamics simulations further corroborated the binding stability, with equilibrated RMSD values (3.38 ± 0.40 Å for nilotinib and 4.56 ± 0.71 Å for lomitapide) and favorable binding free energies (−52.3 ± 4.1 kcal/mol and −49.7 ± 3.5 kcal/mol, respectively) indicating robust interactions under dynamic conditions. The observed reductions in RMSF at key binding site residues, particularly with nilotinib, underscore ligand‐induced rigidity that may prevent conformational changes necessary for TLR2 activation. Additionally, variations in Rg and SASA highlight differential impacts on protein compactness and solvent exposure: nilotinib slightly expands the structure (Rg: 3.06 ± 0.03 nm), while lomitapide enhances compactness (Rg: 2.92 ± 0.04 nm) but increases surface exposure (SASA: 283.99 ± 2.04 nm^2^). Hydrogen bond persistence, though intermittent (averages of 0.46 for nilotinib and 0.87 for lomitapide), supports sustained engagement consistent with the docking poses. To provide a more rigorous thermodynamic foundation and map the energetic hot spots, a dedicated set of MM‐GBSA binding free energy calculations was performed. Both ligands demonstrated highly favorable binding affinities, with ΔG(bind) values of −34.25 ± 0.17 kcal/mol for nilotinib and −37.29 ± 0.22 kcal/mol for lomitapide. Interestingly, while the protonated tertiary amine of lomitapide allows it to form a strong salt bridge with Asp327 (yielding an electrostatic contribution of −18.37 kcal/mol), this formal charge is almost entirely offset by a massive polar desolvation penalty (+18.75 kcal/mol). Consequently, the formal charge of lomitapide does not confer a substantial net affinity advantage, and the binding of both compounds is predominantly driven by van der Waals forces and shape‐complementary hydrophobic packing within the dimer‐interface pocket. Because the 3.04 kcal/mol difference in total binding free energy between the two ligands is smaller than their frame‐to‐frame standard deviations (3.89 and 4.88 kcal/mol, respectively), the two compounds are energetically indistinguishable in these simulations and should not be strictly rank‐ordered based on affinity alone. Per‐residue energy decomposition further identified a shared hot‐spot core at the TLR2‐TLR1 interface. Residues such as Phe325, Phe349, Leu328, and Val348 serve as the primary energetic drivers for both ligands. Notably, this dynamic, ensemble‐averaged energetic profile strongly recapitulates the key contacts identified independently during static docking (e.g., Phe322, Phe325, Asp327, Leu350, Pro352), providing robust mutual validation for the proposed binding mode. Functionally, by stably anchoring into this hydrophobic hot‐spot core, both nilotinib and lomitapide act as steric wedges that lock the TLR2 pocket, preventing the dynamic conformational rearrangements necessary for TLR2‐TLR1 heterodimerization and subsequent signal transduction.

These findings extend prior evidence on TLR2's role in GC, where its overexpression correlates with poor prognosis and drives epithelial proliferation via NF‐κB/MAPK signaling [12, 42, 43]. By targeting this receptor, nilotinib and lomitapide could attenuate autophagy, a known mediator of chemoresistance to agents like cisplatin and 5‐FU, thereby restoring tumor sensitivity [7, 44]. Nilotinib, primarily a BCR‐ABL inhibitor for chronic myeloid leukemia, has demonstrated anti‐inflammatory properties in other malignancies, such as inhibiting p38 MAPK signaling to suppress cytokine expression and monocyte activation in acute myeloid leukemia [45, 46]. This aligns with TLR2's downstream pathways, suggesting nilotinib's repurposing could synergize with its established anti‐cancer effects, including enhanced MHC‐I expression and CD8 + T‐cell activation in colorectal cancer [47]. However, some studies report pro‐inflammatory shifts in chronic myeloid leukemia patients on nilotinib compared to other tyrosine kinase inhibitors, warranting careful evaluation in inflammatory tumor microenvironments [48, 49].

Lomitapide, an inhibitor of microsomal triglyceride transfer protein for hypercholesterolemia, emerges as a novel candidate with emerging anti‐cancer potential through drug repurposing. Preclinical data indicate it induces autophagic cell death via mTOR inhibition in various cancers, including colorectal and glioblastoma models, where it enhances chemotherapy efficacy (e.g., with temozolomide) [50, 51]. In non‐small cell lung cancer, lomitapide overcomes osimertinib resistance by activating autophagy through METTL3 inhibition [52]. While this autophagic induction contrasts with the proposal's aim to suppress protective autophagy in chemoresistant cells, it may contextually promote autophagic overload leading to cell death, particularly in combination therapies [51, 53]. This duality highlights the need for mechanistic validation in GC models, where lipid metabolism alterations could further influence TLR2‐mediated inflammation.

Functionally validating the results, we found that triggering TLR2 with the agonist, Pam3CSK4, increased GC cell proliferation and the expression of autophagy markers, *LC3B‐II, Beclin‐1*, and *p62/SQSTM1*, and reduced apoptosis, while TLR2 blockade using nilotinib and lomitapide or the autophagy inhibitor, chloroquine, reversed these effects and increased apoptotic cell death. These findings are consistent with emerging evidence that TLR signaling in tumor cells can promote pro‐survival pathways and chemoresistance rather than antitumor immunity [11, 43, 54, 55, 56, 57]. West et al. have explored the TLR2‐regulated gene signature related to tumor cell growth in GC and found that elevated TLR2 expression is associated with a growth‐potentiating gene signature in the majority of GC patients predicting poor outcomes, supporting TLR2 as a promising therapeutic target in GC [58]. They also have identified the TLR2‐regulated gene signature of human GC cells corresponds with the upregulation of anti‐apoptotic genes, *BCL2, BCL2A1, CFLAR, TNFAIP3, BIRC3*, and IER3, indicating the close impact of TLR2 activation on apoptosis of GC cells. Consistently, a clinical study of a fully humanized IgG TLR‐2 antibody (OPN‐305) in patients with Myelodysplastic Syndromes (MDS) previously received Hypomethylating Agent (HMA) therapy suggested its potential therapeutic value in patients with MDS [59]. TLR2 signaling has been reported to activate NF‐κB, AKT, and MAPK pathways through *HMGB1* release leading to expansion of chemoresistant cancer stem cells and increased IL‐6 and TGF‐β production in breast cancer [13]. Our results show that Pam3CSK4 increased phosphorylation of NF‐κB p65 and p38 MAPK in GC cells, and the selected TLR2 antagonists (nilotinib and lomitapide) markedly attenuated these signals. Thus, TLR2 appears to engage classic MyD88–NF‐κB/MAPK signaling cascades in GC cells, as reported in other studies. Notably, TLR2 blockade has been reported to restore chemosensitivity by interrupting–survival signaling transferred by NF‐κB in breast cancer cells [13], exactly as we observed in our results in GC cells treated with cisplatin. This suggests that TLR2 signaling functions upstream of key survival pathways that suppress apoptosis, thereby facilitating chemoresistance.

Our findings also highlight a link between TLR2 and autophagy in GC cells. The increased *LC3B* and *Beclin‐1* at both protein and transcript levels was seen with Pam3CSK4‐treated group suggesting that TLR2 activation drives an autophagic response, providing cytoprotection during chemotherapy. This is in line with reports in GC, glioma, and other tumors. For example, Li et al. showed that TLR2 overexpression enhances *LC3‐II* conversion and *Beclin‐1* levels in glioma cells via activating p38 MAPK, thereby promoting proliferation and resistance [10]. Masuda et al. have reported the increased gene expression of autophagy‐related proteins LC3 and Beclin1 in human GC tissues associated with patient's poor survival demonstrating that high autophagy is associated with worse outcomes in GC [60]. Fang et al. have reported that TLR2 can induce autophagy in *Staphylococcus aureus*‐stimulated macrophages via modulating the JNK signaling pathway [61]. Shin et al. found that TLR2 can trigger autophagy via p38 MAPK and ERK signaling pathways upon *Mycobacterium* and *Listera monocytogenes* infection [62, 63]. Thus, collectively, the suppression of autophagy marker induction and sensitizing GC cells to cisplatin by Nilotinib and Lomitapide observed in our results is in line with the concept that blocking autophagy can overcome chemoresistance. Importantly, chloroquine showed similar effects to TLR2 blockade, supporting the concept that TLR2 promotes chemoresistance at least in part by engaging autophagy.

The data related to signaling pathways provide mechanistic insight. Pam3CSK4 treatment increased phospho‐NF‐κB p65 and phospho‐p38 levels, consistent with TLR2's previously reported ability to activate these pathways driving pro‐survival and inflammatory gene expression [13]. Importantly, the elevated p‐p38 we observed is notable, as p38 activity can promote autophagy and inflammatory survival signals. Both compounds we assessed downregulated both phospho‐NF‐κB and phospho‐p38, consistent with directly blocking TLR2 upstream. This is in accordance with the prior findings on nilotinib by Marin‐Rubio et al., demonstrating that it can inhibit p38α and thereby block TLR2/TLR4‐induced cytokine release (TNF‐α, IL‐6) in myeloid cells [45]. They have demonstrated that nilotinib prevents downstream TLR‐induced inflammation via p38 MAPK inhibition. Consistently, our data in GC cells were in line with the mentioned observations and demonstrated that nilotinib can neutralize TLR2 signaling, and it curbs the NF‐κB/MAPK‐driven autophagy that ordinarily protects cells against cisplatin. Intriguingly, the other compound, lomitapide, has been reported to inhibit mTORC1 and stimulate AMPK, which can induce autophagy in cancer cells [53]. Conversely, in this study, we find lomitapide acting antagonistically to Pam3CSK4, suggesting it may also directly interfere with TLR2‐mediated signals.

Overall, our results are consistent with and expand current literature. Tye et al. have reported the upregulated TLR2 by oncogenic *STAT3* in gastric epithelium and the suppression of tumorigenesis using genetic or antibody‐mediated TLR2 blockade in mouse models [42]. They showed the ability of TLR2 to promote epithelial proliferation and survival, and that high TLR2 correlates with poor patient survival. Similarly, we find that TLR2 activation by Pam3CSK4 confers a growth advantage to GC cells under chemotherapy, whereas blocking TLR2 dampens proliferation and triggers apoptosis. The parallel findings have been reported in other malignancies: Marongiu et al. reported that TLR2 drives chemoresistance in breast cancer and that a TLR2 inhibitor, CU‐CPT22, restores doxorubicin sensitivity [13]. Our data underscore that these findings in the context of GC: TLR2 antagonist (using nilotinib or lomitapide) reverse the anti‐apoptotic effects of TLR2 agonist.

In summary, our integrated in silico and experimental approach demonstrates TLR2 as a pivotal driver of autophagy‐mediated chemoresistance in GC. The identification of Nilotinib and Lomitapide as TLR2 inhibitors is especially noteworthy because both are clinically approved, and this potentially allows rapid translation. By binding at the TLR2–TLR1 interface, these drugs appear to allosterically hinder downstream NF‐κB/MAPK signaling, thus suppressing autophagy and reactivating apoptosis in cisplatin‐treated cells. This work, therefore, adds to previous works recognizing TLR2 as a therapeutic target, especially in breast and glioma models, where TLR2 blockade impairs tumor growth and sensitizes to therapy. Ultimately, our results suggest that combining TLR2‐targeted agents with standard chemotherapy could be a promising strategy to overcome refractory disease in GC.

## Author Contributions

**Kuanheng Zheng:** conceptualization, methodology, investigation, writing – original draft, data curation. **Xinjian Lei:** conceptualization, methodology, investigation, writing – original draft. **Bin Lin:** methodology, investigation, formal analysis. **Qianhong Yang:** methodology, investigation, visualization, formal analysis. **Cong He:** investigation, formal analysis. **Jin Gao:** conceptualization, methodology, formal analysis, supervision, investigation, writing – review and editing. **Feng Li:** conceptualization, methodology, investigation, data curation, formal analysis, supervision, project administration, writing – review and editing.

## Conflicts of Interest

The authors declare no conflicts of interest.

## Data Availability

The data sets are available from the corresponding author on reasonable request.
